# Revealing the Interface Characteristic and Bonding Ability of CoCrFeNi High Entropy Alloy/Al Composite by First-Principles Calculations

**DOI:** 10.3390/ma16206692

**Published:** 2023-10-14

**Authors:** Yunzi Liu, Yong Gao, Jian Chen

**Affiliations:** 1 School of Materials and Chemical Engineering, Xi’an Technological University, Xi’an 710021, China; liuyunzi@xatu.edu.cn; 2 Institute of Science and Technology for New Energy, Xi’an Technological University, Xi’an 710021, China; gao_yong@xatu.edu.cn

**Keywords:** first-principle calculation, high entropy alloy composite, interfacial atomic binding evolution, lattice matching

## Abstract

In this work, the interfacial atomic bonding process and atom-matching structure of Al atoms deposited on the crystal plane of CoCrFeNi HEA were investigated by first-principles calculations. The relevant physical parameters, including crystal structure, lattice constants, chemical bonding, and differential charge distribution, were studied in detail. The results showed that the constructed crystal model of CoCrFeNi HEA has a stable structure, and the binding energy of Al atoms deposited constantly on different crystal planes at different sites is less than −16.21 eV, indicating a strong interface bonding ability. With the increase in deposited atoms, the material is subjected to a phase transition from two-dimensional chemical adsorption of Al atoms in a single layer to three-dimensional chemical binding of the bulk. Furthermore, the electron cloud occurred through the interaction of positive and negative charges at the interface, indicating that the charge has been transferred along with a chemical bond between Al and CoCrFeNi atoms. It can be thought that the interface formed a stable structure and possessed low mismatch stress. This work provides a theoretical basis for designing CoCrFeNi series HEA-reinforced Al matrix composites.

## 1. Introduction

The development trend of advanced particle-reinforced metal matrix composites aims at the bottleneck problem of common majors in the fields of national aerospace technology, electronic communication, and transportation [1,2,3,4]. HEA-particle-reinforced Al matrix composites (AMCs), as a kind of advanced composite, show huge potential in many industrial applications [5,6,7,8,9]. As reinforcement particles, HEA possesses broad application fields to fabricate high-performance composites. Compared with traditional reinforcement particles, HEA reinforcements present a smaller difference in thermal expansion coefficient with an aluminum matrix and possess high temperature stability without phase transformation [10,11,12,13,14,15,16,17,18]. Karthik et al. [15] reported that the ultimate tensile stress of the 12 vol.% CoCrFeNi/AA5083 multi-layer composite is more than 1.5 times higher when compared to the AA5083 rod and still maintains 5% tensile elongation. Yang et al. [8] proposed that the addition of AlCoCrFeNi HEA particles would increase the yield strength of the composite by 25.1% while maintaining an acceptable ductility of 18.9%. However, the present investigations lack a theoretical calculation of the interface formation at the atomic scale in HEA-reinforced AMCs.

The material properties of HEAs composed of different elements depend on the geometrical crystal structure of the alloy. The geometric crystal structure is usually determined by the cell parameters, space group, and atomic position in the cell, which determine fundamental factors such as the physical, chemical, and mechanical properties of solid metal materials. For the new multi-principal alloy, the establishment of an appropriate initial setting is necessary to predict the crystal structure and characteristics [19,20,21,22]. First-principle calculation is a suitable method for simulating HEAs with complex structures due to its low costs, high efficiency, and wide range of applications. Gao et al. [20] investigate the NbMoTaTiV HEA by using the DFT method and first-principles calculations, followed by its elastic properties, lattice constants, and electronic properties. Xie et al. [21] reported that they calculated some physical properties of Co_30_Ni_30_Fe_20_Cr_20_ HEA by using first principles calculations, and the results showed that it has a stable structure under normal temperature. At present, CoCrFeNi series HEAs are the most widely and deeply studied ones. Wang et al. [22] thought that Fe, Cr, Co, and Ni have nearly the same atomic size and small mixing enthalpy, and the CoCrFeNi HEAs exhibit high strength, plasticity, and phase stability. Here, the CoCrFeNi HEA was chosen as reinforcement in the Al matrix composite, and the crystal model of the CoCrFeNi HEA was built based on the Density Functional Theory (DFT) method. The CoCrFeNi HEA crystal model was constructed based on cell parameters, spatial groups, and the position of atoms. By setting energy as the condition of adaptive screening, the structural model has been continuously optimized until collection, obtaining a stable structure with lower energy.

In addition, for composite materials, the interface is the “link” between the reinforcement and the matrix, and its structure and formation law directly affect the final structure and comprehensive properties of composites. Thus, evaluating the matching ability and binding state of atoms between the HEA and Al matrix has an important guiding role, which can contribute to understanding the intrinsic properties of the interface on the effect of microstructure and macroscopic mechanical properties in composites. Recently, first-principles calculation has been used to simulate interfacial relationships [15,16,17,18]. After establishing the atomic structure model, Al atoms were randomly deposited on different crystal planes of CoCrFeNi HEA, and the binding energy among atoms was calculated. Subsequently, Al atoms were continued to deposit on the CoCrFeNi model to determine the interface structure evolution process. This process can simplify uncontrollable degrees of freedom, calculate the chemical bonding process between Al atoms and CoCrFeNi HEA, and put forward a novel approach for simulating the evolution process of CoCrFeNi/Al interface bonding. By analyzing the chemical bonding process between interface atoms, the degree of lattice matching between atoms was discussed. Finally, the interfacial bonding ability between the CoCrFeNi high-entropy alloy and the Al matrix was predicted by a first-principles calculation in combination with the DFT model.

## 2. Methodology

CoCrFeNi HEA was selected as a model alloy due to its good comprehensive mechanical [23,24,25] and closer thermal expansion coefficient with that of Al matrix [26], which can be expected to have a good wettability interface. The transition metals have similar radii in CoCrFeNi HEA, and the simulations of the CoCrFeNi HEA model were performed by first-principles calculations. The physical part of the model is the periodic boundary condition of a unit cell, which can be expanded infinitely along the X and Y directions until it becomes a huge unit cell. Thus, it can describe the mechanism of block formation, ensuring that the proportions of these metal atoms in the modeling process are consistent with the experiment results. The Density Functional Theory (DFT) is an efficient calculation method in first principles that can be used to accurately capture the interaction of atoms and electrons. In this work, the formation mechanism of HEA/Al at the interface was explored at the atom and electron levels, which is consistent with the DFT application scale. Furthermore, the CoCrFeNi HEA is considered the base crystal layer, and the Al atoms are randomly deposited on different crystal planes of CoCrFeNi high-entropy alloy atoms. This investigation of the interface bonding ability and structural stability was conducted using Vienna Ab-inito Simulation Package (VASP) software (version number 5.4.1) based on the DFT calculation [27]. VASP software can be used to set the calculation parameters of the optimization script file, such as plane wave truncation energy, geometric model structure, and static calculations about electron distribution. After accurately calculating the force on the atoms and the stress tensor of the system, the position of the atom is relaxed to reach the instantaneous ground state. Furthermore, the Vanderbilt ultra-soft pseudopotential (ultra-soft Vanderbilt pseudopotential, US-PP) and the projection wave method (projector-augmented wave method, PAW) are used for the description of interactions between ion solids and valence electrons. Thus, the number of plane waves required for calculation can be greatly reduced when dealing with systems containing transition metals. In order to obtain high-precision calculation results, the plane wave truncation energy and the K-point grid in the Brillouin region were subjected to a convergence test. After a series of truncation energy test optimizations, the plane wave cutoff energy is set to 450 eV, obtaining the ideal accuracy. Under generalized gradient approximation, the electron exchange correlation is simulated by the Perdew–Burke–Ernzerhof (PBE) functional [28]. The PBE method can correct the position of three-dimensional electron orbits. At the same time, the band gap can be effectively opened by using a suitable number of energy bands, which means that the electrons can overcome the coulomb repulsion between the electrons, leading to the electronic structure returning to the ground state [22]. Additionally, the convergence standard is set to 10^−6^ eV for iterative optimization of electronic structures and 0.1 eV/A for geometric optimization. To ensure the accuracy of the simulation result, the appropriate size should be considered during the modeling process. Finally, the binding energy at different sites on different crystal planes was calculated.

## 3. Results and Discussion

### 3.1. Crystal Structure Model of CoCrFeMnNi HEA

**Figure 1:**
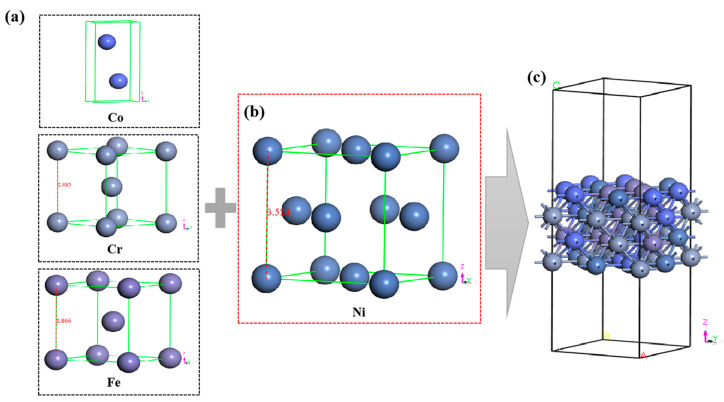
Metal crystal structure and CoCrFeNi HEA model structure: (**a**) Co (light blue balls), Cr (gray balls), Fe (purple balls), (**b**) Ni (dark blue balls), (**c**) and the atomic structure model of CoCrFeNi high entropy alloy with FCC structure and vacuum layer.

Before the simulation of the formation mechanism of the interface, the bonding mode of chemical bonds, and the stability of the interface in the Al matrix composite reinforced by HEA. The modeling idea is to take the HEA as the substrate crystal layer and the matrix Al atom as the bonding atom and constantly add it to the substrate surface to simulate its evolution process. This approach can simplify the uncontrollable degrees of freedom; meanwhile, the chemical bonding process between Al atoms and HEA atoms can be calculated at the atomic level. Therefore, the CoCrFeNi HEA supercell model was simulated at first by first principle calculation. According to the chemical composition of the CoCrFeNi high-entropy alloy with FCC crystal structure in the experiment [29], the metal model was constructed based on the crystal structures of Co (light blue balls), Cr (gray balls), Fe (purple balls), and Ni (dark blue balls), respectively, as shown in Figure 1a,b. Random substitution of Ni atoms in supercells resulted in FCC crystal structures containing 8 Co, Cr, Fe, and Ni atoms, since the Ni crystal structure is FCC and has the largest lattice constant among other metals (3.524 Å), which is similar to the Wang et al. [21] calculated result. The Ni cell was chosen as the basic unit to form a supercell containing 32 Ni atoms, and the Ni atoms were randomly replaced in the Ni supercell to obtain an FCC crystal structure with 8 atoms of Co, Cr, Fe, and Ni, respectively. The lattice constants of a, b, c, α, β, and γ in the space group of this unit cell are 8.6538 Å, 8.6538 Å, 25.4538 Å, 90.0000°, 90.0000°, and 90.0000°, respectively. The chemical composition of each metal element in the obtained model was 25 at.%, which was consistent with the experimental composition structural optimization, and then the model with the lowest energy was found as the optimal structure. Subsequently, a 15 Å true space layer was added to the model. These models were carried out as shown in Figure 1c.

### 3.2. Interfacial Atomic Matching Structure and Binding Energy

**Figure 2:**
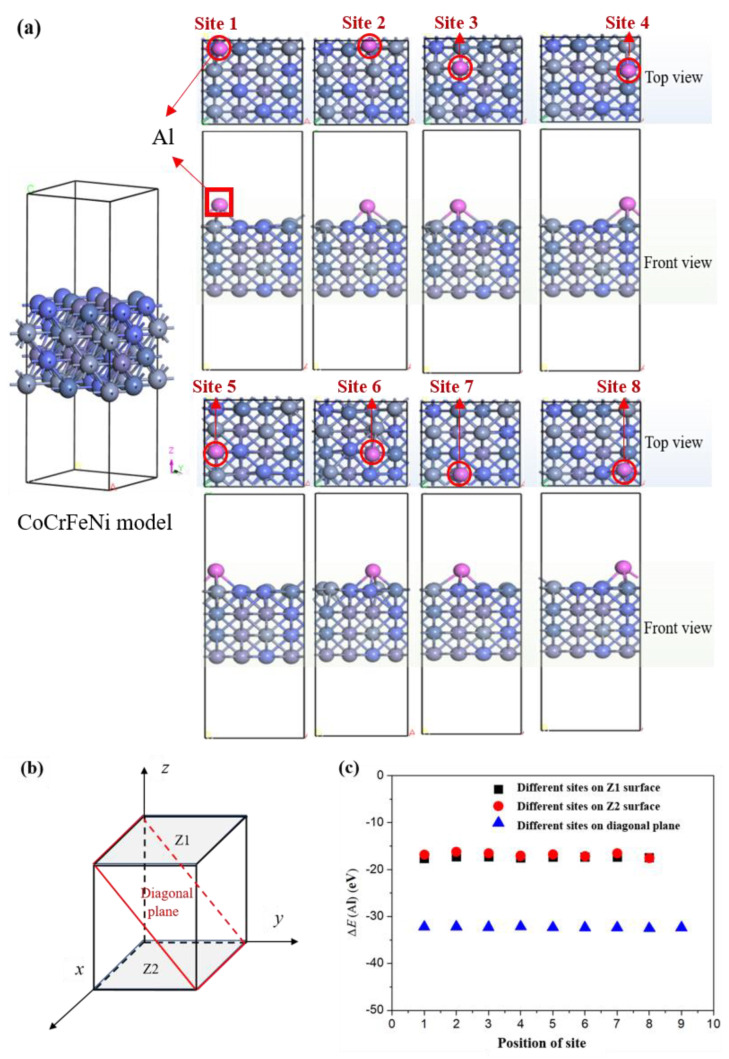
Binding energy and structure diagram of Al atoms (pink balls) at different sites on the surface of CoCrFeNi HEA (the colors of the balls are as same as in Figure 1): (**a**) structure diagram of chemical bonding of Al atoms at different sites on Z1 surface (including top view and front view); (**b**) the crystal structure diagram including Z1 plane, Z2 plane, and diagonal plane; (**c**) binding energy of different sites at Z1 plane, Z2 plane, and diagonal plane.

During the model construction, although the CoCrFeNi HEA has a constant crystal structure and chemical composition, it should be established that different crystal faces, different Al atom binding sites, and different Al atom binding coordination environments can affect the bonding strength, bonding mode, and lattice matching of Al atoms and CoCrFeNi HEA atoms, followed by the influence of the interface properties. To solve these problems, under the same chemical composition and atomic distribution on the same crystal surface circumstances, the effects of different coordination environments and sites on the adsorption of Al atoms were studied, as shown in Figure 2. The CoCrFeNi HEA is considered the base crystal layer, and the Al atoms are randomly deposited on different crystal planes of CoCrFeNi high-entropy alloy atoms. It should be noted that the colors of the balls are as same as in Figure 1, and the pink balls represent Al atoms. Al atoms are placed at different sites to optimize the geometric structure, and then the computational method of quantum theory was used to calculate the total energy of one Al atom that was contained in the cell, as shown in formula (1):

∆*E* = *E*(*substrate* + *Al*) − *E*(*substrate*) − *E*(*Al*) (1)

where ∆*E* represents the change of binding energy before and after Al adsorption, *E*(*substrate + Al*) represents the total energy of the substrate and Al atoms after adsorption of Al atoms, *E*(*substrate*) represents the total energy of the substrate, and *E*(*Al*) represents the total energy of a single Al atom in the ground state, and the calculated value is −3.73 eV. Figure 2a shows that Al atoms are more inclined to chemisorption on the vacancies on the surface of the CoCrFeNi high-entropy alloy with the lowest energy and form chemical bonds with other atoms. Li et al. [19] have reported that the addition of Al can promote the formation of the CoNiFe_0.6_Cr_0.6_ HEA. Moreover, even at the same vacancy, due to the different coordination environment, the strength of chemical bonds is different. This indicates that the direction of the chemical bonding formed by the same vacancy and coordination environment is similar; however, the strength of the bonding formation process still has differences. Subsequently, the binding energies of different sites on the upper and lower surfaces (Z1 and Z2) in the Z direction (see Figure 2b,c) were calculated due to the different directions of load under the service process. In order to further clarify the bonding mode and strength of Al atoms between different crystal planes, the diagonal plane was extracted from the CoCrFeNi HEA during the modeling process (see Figure 2b). The binding energies at different sites are shown in Table 1, corresponding to the marked sites in the top view of Figure 2a. It should be noted that the diagonal plane is a cross section in the model that is bigger than that of the Z1 or Z2 plane, so the binding energies of the diagonal surface have 9 calculated positions of site. Among them, the binding energy on the diagonal plane is far less than that on the Z1 or Z2 plane, because this plane is a tightly packed plane and the atomic coordination environment is not the same with other planes. The results show that the bonding of Al atoms at different sites is basically consistent, indicating that the binding between Al and HEA atoms on the diagonal plane still prioritizes formation on vacancies with the lowest and most stable binding energy structure.

**Figure 3:**
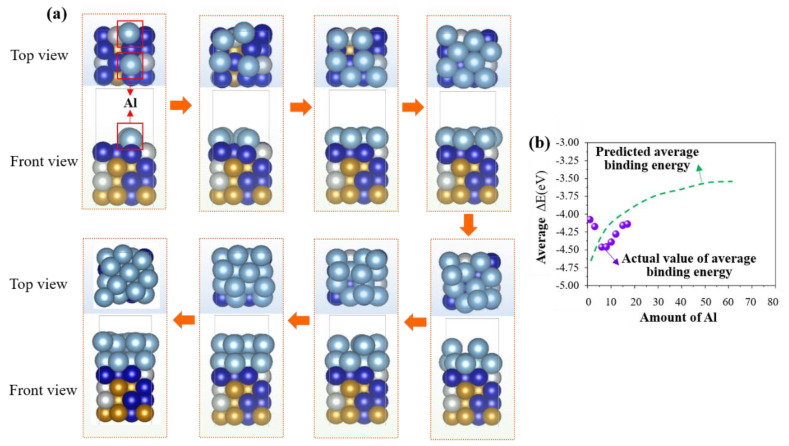
Deposition process and energy change for deposition of Al atoms (the dusty blue balls) on the CoCrFeNi HEA surface (the other colors balls): (**a**) deposition path based on energy change; (**b**) variation of average binding energy with Al content.

Based on the calculation of the binding energy of a single Al atom and a CoCrFeNi HEA atom, it is found that the binding strength is different at each site. However, from an atomic perspective, the formation of an interface involves a chemical bonding process, and the true interface is a phase structure composed of multiple atoms with a certain thickness at the micrometer level. Hence, in order to further investigate the evolution process of the interface, Al atoms were continuously added to the CoCrFeNi HEA substrate. As seen from Figure 3a, the dusty blue balls (inside the red box) represent Al atoms and the other colors balls represent CoCrFeNi HEA substrate. Al atoms form chemical bonds at the lowest energy barrier sites through chemical adsorption. With the adsorption of more and more Al atoms, the adsorption process gradually becomes difficult. The adsorption of Al atoms needs to overcome the adsorption energy barrier of the substrate surface due to the increased coverage. When the coverage rate of Al atoms on the surface of CoCrFeNi HEA reaches 1 (covering a layer), they match well without clustering phenomena among Al atoms through spontaneous fine-tuning. When the interaction energy barrier among Al atoms has reached its maximum value. For the strong interaction release, the material undergoes a phase transition process from two to three dimensions, which means that the two-dimensional single layer of Al atoms is deposited into multiple layers by continuous chemisorption. At the macro-level, a transition layer can be formed by a three-dimensional block chemical bonding transition. The average binding energies of the deposition of Al atoms were calculated in the first and second layers by Formula (2):

(2) $$\Delta \bar{E}=\frac{E(\mathrm{subtrate}\;+\;\mathrm{nAl})\;-E(\mathrm{subtrate})\;-\mathrm{nE}(\mathrm{Al})}{n}\;$$

where E(subtrate+nAl) represents the total energy of the substrate and Al atoms after absorption n Al atoms. The continuous increase in average adsorption energy belongs to a homogeneous reaction process, and external energy is required to complete this nonspontaneous process during interface formation. This is also consistent with the viewpoint reported by Cui et al. [30]. The small purple balls represent the required average binding energy, which was calculated by the DFT method. It should be noted that this calculation suffers from limitations in computing power; the green dashed line represents the predicted value of the binding energy required for calculating and simulating more Al atoms, as shown in Figure 3b. Apparently, as the Al atoms increase, the green dashed line becomes smooth and slow, indicating that the simulation results will tend to form a stable transition layer. This suggests that the energy calculations of the first and second layers have shown a phase transition trend from a two-dimensional plane to a three-dimensional block material, followed by good lattice matching between Al and HEA atoms.

**Figure 4:**
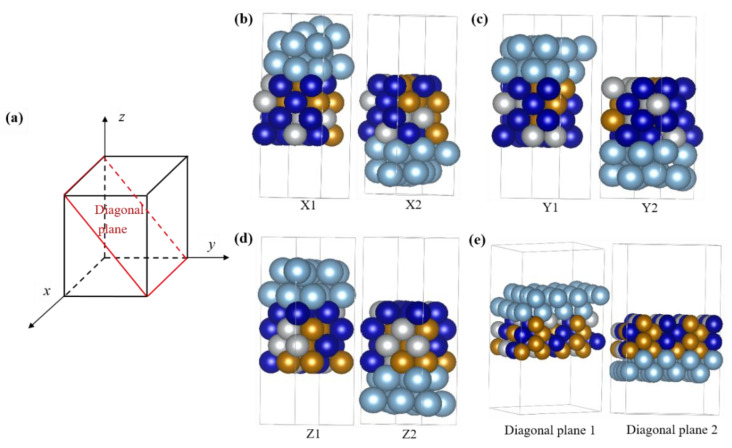
Atomic structure diagram for deposition of two-layer Al atoms on different crystal planes of CoCrFeN HEA(the colors of balls as same as Figure 3): (**a**) the crystal structure diagram; (**b**) X1 plane and X2 plane corresponding to the front and rear surfaces of the X-axis; (**c**) Y1 plane and Y2 plane corresponding to the left and right surfaces of the Y-axis; (**d**) Z1 plane and Z2 plane corresponding to the upper and lower surfaces of the Z-axis; (**e**) diagonal plane 1 and diagonal plane 2 corresponding to the upper and lower surfaces of the diagonal plane.

Furthermore, the matching between Al atoms and different CoCrFeNi HEA crystal planes was studied, as shown in Figure 4. Figure 4a shows the crystal structure diagram of CoCrFeNi HEA, including 8 crystal planes, and Figure 4b displays the atomic matching structure of the 8 crystal plans combined with two layers of Al atoms (the colors of balls as same as Figure 3), respectively. Obviously, although these crystal planes have different surface compositions, different atomic distributions, different coordination environments, and even different crystal face indices, they all have good lattice matching with Al atoms, resulting in a reduction of mismatch stress at the interface between reinforcement and matrix.

**Figure 5:**
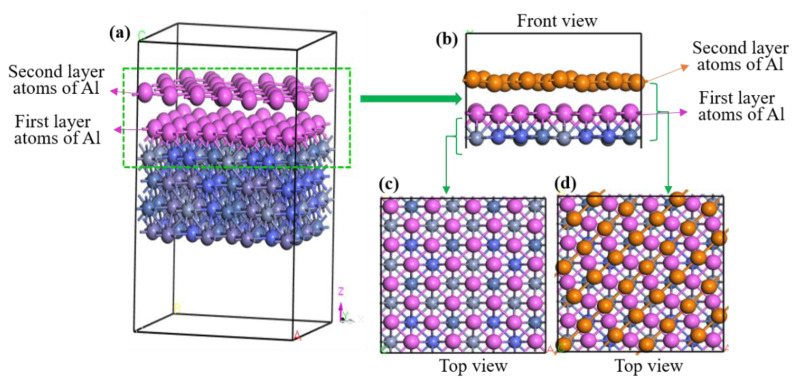
Structure diagram of deposition of lattice matching for two-layer Al atoms on the CoCrFeNi HEA surface: (**a**) three-dimensional image (two layers pink balls represent Al atoms); (**b**) front view; (**c**) top view of single-layer Al atomic deposition; (**d**) top view. Noted that the first layer and second layer Al atoms were shown as pink and saffron yellow in front view and top view, respectively.

Based on the calculation results of the chemical binding of multi-layer Al atoms, the lattice matching state between the Al matrix and CoCrFeNi HEA is further studied. As learned from Figure 5a, the two layers pink balls represent Al atoms. For a clearer distinction, the first layer and second layer Al atoms were shown as pink and saffron yellow, respectively. It can be seen that the first layer of Al atoms has strong bonding with the CoCrFeNi HEA. Figure 5b shows the main view of the binding of two layers of Al atoms with adjacent CoCrFeNi HEA atoms (change the color of the Al atom in the second layer to orange for clarity). Figure 5c,d, respectively, show the top views of the matching state between the first- or second-layer Al atoms and the CoCrFeNi HEA substrate atoms. Evidently, the first layer of Al atoms is located at vacancies in the CoCrFeNi HEA atoms, suggesting a very good matching effect. Different from the first layer, the second layer of Al atoms is mainly located on the bridge sites, which can still be thought of as having a good degree of matching (see Figure 5d).

### 3.3. Chemical Bonding and Charge Distribution

**Figure 6:**
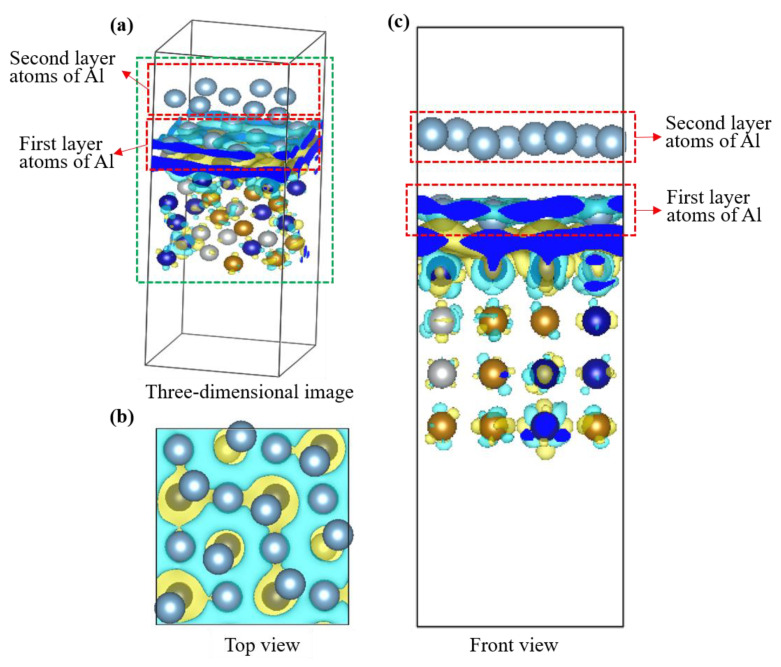
Differential charge distribution of two-layer Al atoms (the dusty blue balls) bonding CoCrFeN HEA atoms (the other colors balls) combined with lattice matching: (**a**) three-dimensional image, (**b**) top view, (**c**) front view.

Figure 6 shows the differential charge distribution diagram of the binding layer between Al atoms and the HEA atom substrate, where the blue represents positive charge and the yellow represents negative charge. The interaction between the blue positive charge and the yellow negative charge has formed an electron cloud distribution, indicating that the charge has been transferred and a chemical bond has been formed between the Al atoms and the CoCrFeNi atoms. It can be confirmed that the formation of the interface is a chemical bonding process, indicating a relatively stable interface.

## 4. Conclusions

In this work, first-principles calculations are used to investigate the interface binding energy between CoCrFeNi HEA and Al atoms, optimize their structures, and simulate the matching evolution process of the interface. By depositing Al atoms on the surface of the CoCrFeNi HEA model, the matching evolution process between atoms was analyzed, while the binding energy of interface atoms and the charge distribution of chemical bonds were combined to simulate the formation process of the interfaces. The main conclusions can be drawn as follows:

(1) An optimization CoCrFeNi HEA unit cell model with an FCC crystal structure was established by first-principles calculations and calculated the total energy of a single Al atom, which is −3.73 eV;
(2) When the Al atoms are deposited at different sites on different crystal planes of HEA, the binding energy values are less than −16.21 eV. The interfacial atomic matching evolution process shows very little mismatch stress, tending to form a stable three-dimensional transition layer between the reinforcement and the matrix in the CoCrFeNi/Al composite;
(3) The electron cloud distribution through charge transfer reveals the strong chemical bond formation and confirms that the interface formation is a chemical bonding process, indicating a strong interfacial bonding ability.

## Figures and Tables

**Table 1:** Binding energy at different sites on different crystal planes (eV).

Sites	1	2	3	4	5	6	7	8	9
Z1	−17.67	−17.33	−17.32	−17.52	−17.36	−17.34	−17.35	−17.43	-
Z2	−16.86	−16.21	−16.46	−17.05	−16.72	−17.14	−16.50	−17.52	-
Diagonal plane	−32.25	−32.19	−32.26	−32.15	−32.29	−32.36	−32.34	−32.49	−32.35

## Data Availability

Not applicable.
